# Novelty Before or After Word Learning Does Not Affect Subsequent Memory Performance

**DOI:** 10.3389/fpsyg.2019.01379

**Published:** 2019-06-27

**Authors:** Davina Biel, Nico Bunzeck

**Affiliations:** Institute of Psychology I, University of Lübeck, Lübeck, Germany

**Keywords:** novelty, memory enhancement, recognition memory, recollection, familiarity

## Abstract

In humans, exposure to novel images and exploration of novel virtual environments before the encoding of words improved subsequent memory performance. Animal studies revealed similar effects of novelty, both before and after learning, and could show that hippocampus-dependent dopaminergic neuromodulation plays an important role. Here, we further investigated the effects of novelty on long-term memory in humans using a novel paradigm employing short sequences of nature movies presented either before or at two time points after learning of unrelated words. Since novelty processing is associated with a release of dopamine into the hippocampus, we hypothesized that novelty exposure primarily affects hippocampus-dependent memory (i.e., recollection) but not hippocampus-independent memory (i.e., familiarity). We tested 182 healthy human subjects in three experiments including a word-learning task followed by a 1-day delayed recognition task. Importantly, participants were exposed to novel (NOV) or familiar movies (FAM) at three time points: (experiment 1) directly after encoding of the word list, (experiment 2) 15 min after encoding, (experiment 3) 15 min prior to encoding. As expected, novel movies were perceived as more interesting and led to better mood. During word recognition, reaction times were faster for remember as compared to familiarity responses in all three experiments, but this effect was not modulated by novelty. In contrast to our main hypothesis, there was no effect of novelty – before or after encoding – on subsequent word recognition, including recollection and familiarity scores. Therefore, an exposure to novel movies without an active task does not affect hippocampus-dependent and hippocampus-independent long-term recognition memory for words in humans.

## Introduction

A few recent studies in humans have shown that the exposure to novelty before a learning phase improves subsequent memory (Fenker et al., 2008; Ballarini et al., 2013; Schomaker et al., 2014). For instance, the presentation of novel images before a word-learning task enhanced free recall and recollection-based memory (Fenker et al., 2008); recall rates of words could be enhanced through an active exploration of a novel virtual environment before the learning phase (Schomaker et al., 2014); and already familiar scene images were subsequently better recognized when they were presented in the context of novel images as compared to a context with very familiar images (Bunzeck and Düzel, 2006). These observations in humans largely fit to animal studies, which also show that long-term memory is not only promoted through novelty exploration before – but also after – learning (Moncada and Viola, 2007; Wang et al., 2010). To our knowledge, however, such a positive effect of novelty after learning has not been reported in humans yet.

The processing of novel information recruits the dopaminergic mesolimbic system. Specifically, the hippocampal-VTA loop model suggests that the medial temporal lobe (including the hippocampus and surrounding cortex) detects novelty by comparing incoming with predicted information (Lisman and Grace, 2005; Lisman et al., 2011). The resulting neural novelty signal is then send to the dopamine (DA) neurons in the substantia nigra/ventral tegmental area (SN/VTA) *via* a polysynaptic path, including the subiculum, nucleus accumbens, and ventral pallidum. In turn, DA neurons back-project to the hippocampus, where DA is involved in several forms of learning. For instance, the late phase of hippocampal long-term potentiation (LTP) is DA dependent (O’Carroll and Morris, 2004; Granado et al., 2008), and injections of DA agonists into the hippocampus improve memory processes in rats (Packard and White, 1991). The role of the SN/VTA, hippocampus and also DA in novelty processing has been underlined in functional imaging studies in humans (Chowdhury et al., 2012; Bunzeck et al., 2014), and therefore, the hippocampal-VTA model helps to explain the beneficial effects of novelty on long-term memory. More direct evidence comes from Wang et al. who could show in rats that novelty exploration after spatial encoding improves long-term place-memory (i.e., at a behavioral level), and this effect was blocked by D1/D5 receptor antagonists (Wang et al., 2010).

Recent studies have shown that novelty also activates the noradrenergic system, which co-releases noradrenaline and DA into the hippocampus. Therefore, hippocampal DA has two sources (McNamara and Dupret, 2017; Duszkiewicz et al., 2019), and novelty-dependent activation of the noradrenergic locus coeruleus also drives hippocampus-dependent learning (Kempadoo et al., 2016) and consolidation of everyday memory (Takeuchi et al., 2016) *via* dopaminergic neuromodulation.

Recognition memory in humans is often investigated using the remember/know paradigm (Tulving, 1985). It assumes that recognition can be associated with specific details or associations of the encoding episode (i.e., recollection), or in the absence of such recollective experience (i.e., familiarity). Further support for such a dual process (Yonelinas et al., 1996, 2010) comes from functional imaging studies, suggesting that different regions of the medial temporal lobe are involved in recognition memory processes depending on task demands and type of information (Diana et al., 2007). In particular, while the hippocampus and posterior parahippocampal gyrus are closely associated with recollection, the anterior parahippocampal gyrus is more associated with familiarity (Diana et al., 2007). Therefore, the hippocampus appears to be more critical for recollection but not for familiarity (Yonelinas et al., 2010). Furthermore, reaction times (RTs) for items that are associated with recollection are typically faster as compared to familiarity, which further indicates different processes (Dewhurst et al., 2006; Rotello and Zeng, 2008; Gimbel and Brewer, 2011). Together, the remember/know paradigm provides a good tool to differentiate hippocampus-dependent from hippocampus-independent memory performance.

In animal studies, the effects of novelty on learning are typically investigated by using an active exploration of a new vs. familiar environment (Li et al., 2003; Davis et al., 2004; Moncada and Viola, 2007; Wang et al., 2010). Studies in humans, however, often used static images (Fenker et al., 2008) or virtual environments (Schomaker et al., 2014) before a word-learning task or static images in the context of learning (Bunzeck and Düzel, 2006). In the case of Schomaker et al. (2014) and Fenker et al. (2008), the novelty presentation was 5 min long, which was based on prior observations in animals suggesting that a 5 min novelty exploration is sufficient to facilitate LTP (Li et al., 2003); in the case of Bunzeck and Düzel (2006), however, several repeating learning contexts with novel and familiar items were approx. 6 min long, suggesting that a limitation of 5 min might not necessarily be justified. Indeed, in a study with rats, the animals stayed in the novel environment for about 15 min, which led to a reinforcement of early- to late-LTP (Straube et al., 2003). And, finally, long-term memory in school children could be promoted by a 20-min novel science lesson 1 h before or after story reading (Ballarini et al., 2013). This latter finding also demonstrates that the beneficial effects of novelty have practical implications, and therefore, a thorough understanding of the underlying processes is important.

In this study, we investigated (1) whether other forms of novelty stimulation drive word-learning and (2) whether a critical time-window exists in humans (as seen in animal studies). Therefore, we employed a novel paradigm including the presentation of short (13 min) nature movies (1) shortly after, (2) 15 min after, and (3) before encoding of a word list, and tested long-term memory for these unrelated words after a 1-day delay (based on the assumption that DA affects late LTP and therefore long-term memory; Wittmann et al., 2007; Lisman et al., 2011). We expected a positive effect of novelty before and after word-learning that is particularly pronounced for hippocampus-dependent recollection. Moreover, we expected faster RTs for recollection as compared to familiarity, which might be further modulated by novelty (i.e., even faster recollection when a word was learned before or after novelty presentation). Finally, we expected novel movies to be more interesting than repeatedly presented familiar ones and a positive effect of novel movies on mental states (i.e., the novel movies lead to higher attentional states, including wakefulness, compared to familiar movies). The latter hypotheses are based on previous studies, showing high novelty preferences in particular for natural scenes as compared to faces or geometric figures (Park et al., 2010).

## Materials and Methods

### Participants

In total, 192 healthy, right-handed, German-speaking participants were recruited for three experiments. Five participants were excluded because their behavioral performance (including hit rates and RTs) was more than 3 standard deviations (SD) above the mean, one subject did not return on day 2, and four were excluded for technical reasons or other problems. Finally, 182 participants were randomly assigned into three experimental groups (NOV) and three control groups (FAM). In experiment 1, 61 participants were tested (NOV = 32 participants, FAM = 29 participants; mean age = 23.07 ± 3.62 years, 44 women); in experiment 2, 60 participants (NOV = 31 participants, FAM = 29 participants; mean age = 22.32 ± 3.07 years, 51 women) were tested; and in experiment 3, 61 participants (NOV = 30, FAM = 31; mean age 22.69 ± 3.27 years, 51 women) were tested (Table 1). All subjects were recruited through the database of the University of Lübeck (Greiner, 2015) and signed a written informed consent. For compensation, participants received either credits points (psychology students only) or 10 € per hour (i.e., in total between 10 and 15 €). The study was approved by the local ethics committee of the University of Lübeck, Germany, and in accordance with the Declaration of Helsinki.

**Table 1:** Demographics.

	Experiment 1				Experiment 2				Experiment 3			
	Directly after encoding				15 min after encoding				15 min prior to encoding			
Groups	NOV		FAM		NOV		FAM		NOV		FAM	
*n*	32		29		31		29		30		31	
Age	23.31 (±3.74)		22.79 (±3.53)		23.45 (±3.49)		21.10 (±1.95)		22.83 (±3.25)		22.55 (±3.34)	
Sex	♀	♂	♀	♂	♀	♂	♀	♂	♀	♂	♀	♂
*n*	22	10	22	7	24	7	27	2	25	5	26	5

### Procedure

**Figure 1:**
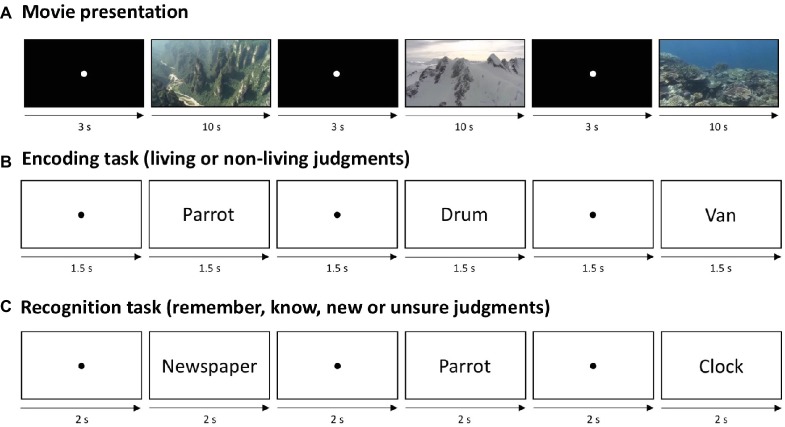
Experimental design. **(A)** In all three experiments, subjects watched either novel (NOV group) or five repeating (FAM group) movie clips for 13 min. The movies were presented directly after (experiment 1), 15 min after (experiment 2) or 15 min prior to (experiment 3) encoding. **(B)** During encoding, participants classified nouns into “living” vs. “non-living” by button presses. **(C)** On the second day, all 100 words from the encoding phase were presented intermixed with 100 new words, and participants classified them into “remember”, “know”, “new,” or “unsure.”

The experiments took place on 2 consecutive days. On day 1, participants performed an encoding task in which they classified words into living vs. non-living by button presses. In total, 50 living and 50 non-living German nouns were randomly presented on a white computer screen (13 inches) in black letters (Arial, 30 point) for 1.5 s followed by a fixation point (also 1.5 s; Figure 1B). Participants were asked to respond as quickly and as accurately as possible. In case of an omission or incorrect response, a corresponding feedback appeared on the screen (i.e., “too slow” or “incorrect response”). This encoding phase took approx. 5 min.

In all three experiments, a 13-min movie phase (Figure 1A) preceded or followed the encoding task. Here, participants were instructed to carefully watch 10 s nature movie sequences (no other task was required during the movie presentation), separated by a 3 s white fixation point on a black screen.

The movies depicted different nature settings from five regions including Africa, America, Asia, Europe, and Oceania. In order to avoid a drop of attention – which could occur using only one longer movie sequence – movies were randomly presented with a duration of 10 s each. The sequences did not show any humans. In addition, scenes with strong emotional content were avoided to prevent high arousal (e.g., hunting predators). There was no relationship in terms of content between movies and words.

For the NOV groups, 60 novel sequences were presented, while the FAM groups watched three different movies, which were repeated 20 times. Since only three movies were shown to the FAM groups, a separate familiarization phase was not implemented.

For the three experiments, the novelty phase was implemented at different time points: directly after encoding (experiment 1), 15 min after encoding (experiment 2), and 15 min prior to encoding (experiment 3). During the 15-min break, participants were instructed to quietly wait on their seats. Directly after watching the movies, participants were instructed to rate the previously presented movies on an interval scale reaching from very uninteresting to very interesting. Further, shortly before and after the exposure to the movies, participants filled out a multidimensional mental state questionnaire (Mehrdimensionale Befindlichkeitsfragebogen, MDBF) covering: good mood/bad mood, wakefulness/tiredness, and calmness/restlessness.

On the second day of the experiment, participants performed a modified version of the remember/know recognition memory paradigm (Tulving, 1985). Here, the 100 words from the encoding task were intermixed with 100 new words (50 living and 50 non-living words) and randomly presented at the center of a screen. Participants were instructed to categorize these 200 words into “remember” (i.e., remembering something specific about reading the word at encoding), “know” (recognizing the word without any recollective experience), “new,” or “unsure” (Figure 1C) *via* button presses. Participants had 4 s in total for making a judgment (i.e., 2-s word presentation followed by a fixation point for 2 s).

Following previous studies (e.g., Fenker et al., 2008), participants were not tested on the novel movie sequences, and therefore, they were not informed about a possible relation between the movies and the word-related memory task. All words were taken from a pool of words and randomly assigned to experimental conditions. Thus, there was no preselection or assignment of words to certain groups or conditions.

The experiment was programmed with Psychophysics Toolbox 3.0.10 (Brainard, 1997) and Matlab (R2014b version) software.

Since the movie rating scale and the MDBF were not implemented from the beginning of the study, in experiment 1 only 55 out of 61 participants filled out the rating scale. From these, the first 37 participants completed the scale on day 2 instead day 1, after finishing the recognition task. In experiments 2 and 3, all participants rated the movies directly after presentation. For the MDBF, 105 out of 182 participants completed the questionnaire: 24 out of 61 participants in experiment 1, 23 out of 60 in experiment 2 and 58 out of 61 in experiment 3.

### Statistical Analysis

For the encoding task, hit rates (HRs) were analyzed as the proportion of correct answers (relative to all possible correct answers). For the subsequent recognition task, corrected hit rates (cHRs) of remember (cHR-remember) and know (cHR-know) answers were defined as follows:

$$cHR=\frac{\mathrm{correct}\;\mathrm{hits}}{\mathrm{possible}\;\mathrm{correct}\;\mathrm{hits}}-\frac{\mathrm{false}\;\mathrm{alarms}}{\mathrm{possible}\;\mathrm{false}\;\mathrm{alarms}}\text{.}$$

Moreover, RTs were analyzed for the encoding and recognition task. Here, within each subject, RTs of 2 SD above and below the subject’s mean were excluded, and the remaining trials were averaged for subsequent between-subjects analyses.

To ensure that groups did not differ at baseline, HR and RT for day 1 (encoding task) were investigated using two-way ANOVAs (3 × 2) with the between-subject factors *time point* of movie presentation (experiment 1: directly after encoding vs. experiment 2: 15 min after encoding vs. experiment 3: 15 min prior to encoding), and *novelty* (NOV vs. FAM). The effects of novelty on memory performance for day 2 (recognition task) were investigated using a three-way mixed-design ANOVA (3 × 2 × 2) with the between-subject factors *time point* (experiments 1, 2, and 3, as above), *novelty* (NOV vs. FAM), and the within-subject factor *memory* (cHR-remember vs. cHR-know or RT-remember vs. RT-know).

The relationship between novelty and movie rating was analyzed using a two-way ANOVA (3 × 2). Further, a 3 × 2 × 3 MANOVA with the between-subject factors *time point* and *novelty* and the within-subject factor *inner state* (*good mood/bad mood vs. wakefulness/tiredness vs. calmness/restlessness*) was conducted for the mental state questionnaire. Finally, *post hoc t*-tests were used when applicable with a Bonferroni adjusted alpha level of *p* = 0.025 (0.05/2). All statistical analyses were performed using IBM SPSS Version 24.

## Results

On average, participants discriminated living vs. non-living nouns with a mean HR of 0.96 ± 0.02 (minimum 0.9, maximum 1; range 0–1). The mean RT was 884 ± 105 ms. A 3 × 2 ANOVA with the factors *time point* and *novelty* on HRs and RTs revealed no main effects and no interactions [HRs: *novelty: F*(1,176) = 0.692, *p* = 0.406, partial *η*^2^ = 0.004; *time point: F*(2,176) = 1.664, *p* = 0.192, partial *η*^2^ = 0.019; *novelty × time point: F*(2,176) = 0.349, *p* = 0.706, partial *η*^2^ = 0.004; RTs: *novelty: F*(1,176) = 2.373, *p* = 0.125, partial *η*^2^ = 0.013; *time point: F*(2,176) = 0.348, *p* = 0.706, partial *η*^2^ = 0.004; *novelty × time point: F*(2,176) = 0.587, *p* = 0.557, partial *η*^2^ = 0.007].

For the recognition memory task, a 3 × 2 × 2 ANOVA on cHR-remember and cHR-know revealed no main effects [*memory: F*(1,176) = 0.295, *p* = 0.587, partial *η*^2^ = 0.002; *time point: F*(2,176) = 0.216, *p* = 0.806, partial *η*^2^ = 0.002; *novelty: F*(1,176) = 0.510, *p* = 0.476, partial *η*^2^ = 0.003] and no interactions [*memory × time point: F*(2,176) = 1.972, *p* = 0.142, partial *η*^2^ = 0.022; *memory × novelty: F*(1,176) = 0.258, *p* = 0.612, partial *η*^2^ = 0.001; *memory × time point × novelty*: *F*(2,176) = 0.348, *p* = 0.706, partial *η*^2^ = 0.004].

**Figure 2:**
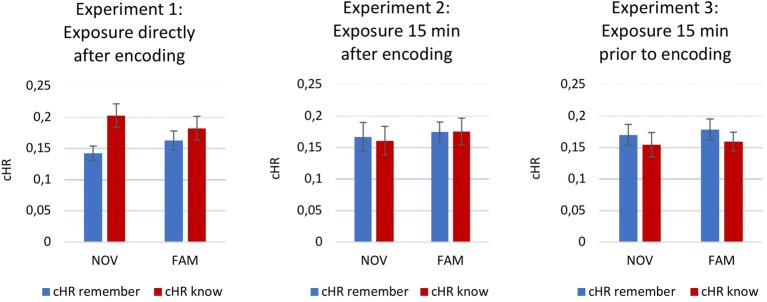
Recognition memory performance. Corrected hit rate (cHR) for remember and know for all three experiments. Error bars indicate ±1 standard error of the mean (SEM).

Subsequently, two separate 3 × 2 ANOVAs were conducted for both, cHR-remember and cHR-know. Again, no significant main effects or interactions could be observed for cHR-remember [*time point: F*(2,176) = 0.990, *p* = 0.374, partial *η*^2^ = 0.011; *novelty: F*(1,176) = 0.790, *p* = 0.375, partial *η*^2^ = 0.004; *novelty × time point: F*(2,176) = 0.092, *p* = 0.912, partial *η*^2^ = 0.001] or cHR-know [*time point: F*(2,176) = 1.736, *p* = 0.179, partial *η*^2^ = 0.019; *novelty: F*(1,176) = 0.000, *p* = 0.986, partial *η*^2^ = 0.000; *novelty × time point: F*(2,176) = 0.429, *p* = 0.652, partial *η*^2^ = 0.005]. Figure 2 depicts cHR-remember and cHR-know for all three experiments and groups.

**Figure 3:**
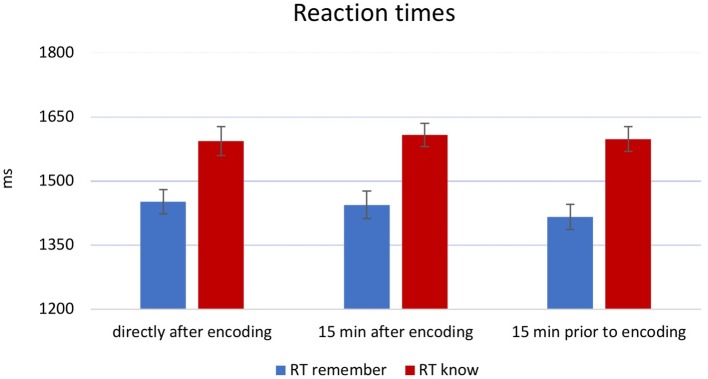
Results for RTs at retrieval. Overall, reaction times (RTs) were faster for “remember” than “know” responses (main effect), but there was no significant effect of novelty. For display purposes, groups were combined. Error bars indicate ±1 SEM.

A 3 × 2 × 2 ANOVA on RTs (during recognition) showed a main effect of *memory* [*F*(1,174) = 127.61, *p* < 0.001, *η*^2^ = 0.42], but no other main effects [*time point: F*(2,174) = 0.102, *p* = 0.903, partial *η*^2^ = 0.001; *novelty: F*(1,174) = 0.078, *p* = 0.781, partial *η*^2^ = 0.00]. *Post hoc* analysis revealed significantly faster “remember” responses in contrast to “know” responses (Figure 3). There was no significant interaction between *novelty* and *time point* [*F*(1,174) = 0.097, *p* = 0.755, partial *η*^2^ = 0.001; *F*(2,174) = 0.780, *p* = 0.460, *η*^2^ = 0.009]. Finally, a *memory × time point × novelty* interaction also did not reach significance [*F*(2,174) = 0.228, *p* = 0.796, partial *η*^2^ = 0.003].

**Figure 4:**
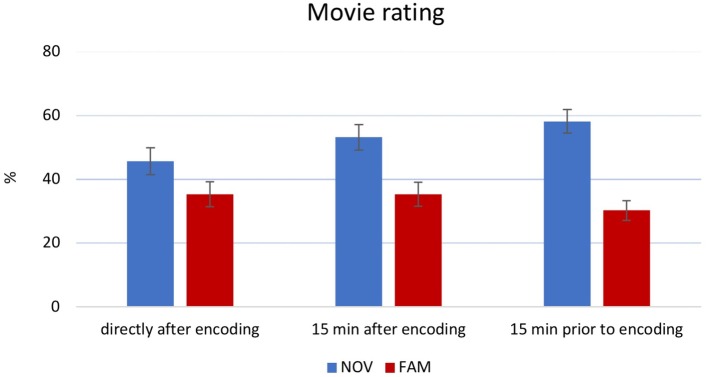
Main effect of movie rating. Participants of the NOV group (mean 52.51%) rated movies more positive than participants in the FAM group (mean 33.52%). Error bars reflect ±1 SEM.

A 3 × 2 ANOVA on movie ratings revealed a main effect of *novelty* [*F*(1,170) = 36.59, *p* < 0.001, partial *η*^2^ = 0.177]. *Post hoc* analysis showed that novel movie clips were rated more positive as compared to the familiar movie clips (Figure 4). The *novelty × time point* interaction was not significant [*F*(2,170) = 2.699, *p* = 0.07, partial *η*^2^ = 0.031].

**Figure 5:**
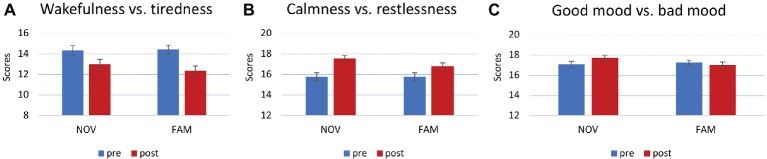
Mental state ratings after movie presentation. **(A)** Main effect for wakefulness vs. tiredness. Higher values represent wakefulness, lower values tiredness. **(B)** Main effect for calmness vs. restlessness. Higher values represent calmness, lower values restlessness. **(C)** Interaction between good mood vs. bad mood and group. Higher values represent good mood, lower values bad mood. Increase of good mood in the NOV group, no change in the FAM group.

A 3 × 2 × 3 MANOVA on ratings of mental states (MDBF) showed main effects of *calmness/restlessness* [*F*(1,99) = 24.536, *p* < 0.001, partial *η*^2^ = 0.199] and *wakefulness/tiredness* [*F*(1,99) = 42.041, *p* < 0.001, partial *η*^2^ = 0.298; Figures 5A,B]. *Post hoc* paired *t*-tests revealed that in both, the NOV and the FAM group, scores of wakefulness decreased and calmness increased from pre- to post-inner state assessment [NOV: *t*(52) = 3.587, *p* = 0.001; *t*(52) = −5.571, *p* < 0.001; FAM: *t*(51) = 6.05, *p* < 0.001; *t*(51) = −2.674, *p* = 0.01]. Finally, a statistically significant interaction was observed between *novelty × good mood/bad mood* [*F*(1,99) = 6.773, *p* = 0.011, partial *η*^2^ = 0.064; Figure 5C]. *Post hoc* analysis (paired *t*-tests) for the NOV and FAM group separately – each averaged across experiments – showed that good mood ratings of the NOV group increased [*t*(52) = −4.072, *p* < 0.001], while good mood ratings of the FAM group did not change [*t*(51) = 0.865, *p* = 0.391].

## Discussion

We investigated whether the exposure to novel nature movies before or after encoding of a word list can improve subsequent long-term memory performance. Although novel (in contrast to repeated) movies were rated as more interesting and had a more positive effect on mental states, they did not improve long-term memory in any of our three experiments. Specifically, novel movies right after, 15 min after or before encoding did not affect familiarity- or recollection-based recognition memory scores. Our findings suggest that an exposure to novelty without an active task is not sufficient in order to promote subsequent long-term memory. In the following, we will discuss several explanations of our null finding, and conclude that a sense of agency with the novel material appears to be necessary in order to induce a positive effect on learning.

On the basis of previous work, we hypothesized that novelty promotes subsequent long-term memory since it activates the mesolimbic and noradrenergic system leading to DA release into the hippocampus (Lisman and Grace, 2005; Lisman et al., 2011; Duszkiewicz et al., 2019). Therefore, a rather physiological explanation for our null finding is that the employed stimulus material (video sequences) simply did not lead to the cascade of mesolimbic and noradrenergic activity and subsequent DA releases. While there is sufficient evidence that novel scene images activate the SN/VTA, striatum, and hippocampus (Bunzeck and Düzel, 2006; Zaehle et al., 2013; Bunzeck et al., 2014; Herweg et al., 2018), it remains unclear whether the same is true for novel nature movie sequences. Indeed, several forms of novelty have been dissociated previously, further indicating conceptual differences. Specifically, item novelty, contextual novelty and spatial novelty might differ from surprise and contextual deviance in terms of underlying processes and associated cognition. An elegant overview of these and related concepts can be found in Schomaker and Meeter (2015).

Along the same lines, novelty can be interpreted in absolute and relative terms, in the sense that expectations about upcoming information drive novelty processing. For instance, within the medial temporal lobe, novelty signals adaptively scale according to expected contextual probabilities of new and familiar events (Bunzeck et al., 2010). In other words, when cues predict a familiar but contextually novel item with equal probability, the familiar item leads to similar neural activity as compared to a novel item (in another context). Therefore, continuously presented familiar and novel movie sequences may have led to similar mesolimbic neural activity due to its adaptive properties, and the repetitive and predictive character of our paradigm. On the other hand, novel movies were, on a subjective level, rated as more interesting than familiar ones (Figure 4), and this was paralleled by a more positive mental state. Specifically, novel movies induced a better mood as compared to familiar ones. Although this was expected, and is in line with previous findings (Park et al., 2010), there was no apparent effect on subsequent or prior word-learning, which might relate to the relatively small effects of novelty on mood (Figure 5C). Together, despite a positive subjective effect (interest and mood), it appears possible that the presented novel movie sequences did not lead to neural activity within the mesolimbic system. This hypothesis, however, can only be supported by future studies using fMRI or other appropriate techniques.

A more likely explanation for our null finding of novelty on memory relates to differences in task requirements. In contrast to our experiment, subjects in other studies were actively engaged with the novel material. For instance, in Fenker et al. (2008), subjects had to make an indoor/outdoor discrimination on scene images, which, in the case of novel images, enhanced recollection and free recall of subsequently learned words. In Schomaker et al. (2014), humans actively explored a novel virtual environment, which also enhanced free recall of a subsequently learned word list. In children, the active and attentive participation in a novel science class before or after reading a story improved subsequent memory (Ballarini et al., 2013). Such active engagement with the novel stimulus material is comparable to animal studies, in which rodents are allowed to actively and freely explore a novel (vs. familiar) environment; this promotes hippocampal LTP and also drives learning and memory (Li et al., 2003; Ballarini et al., 2009; Wang et al., 2010). Further support and possible explanations of a close link between active behavior and learning comes from human studies. They indicate that a sense of agency, for instance through active choices during learning, promotes subsequent declarative long-term memory, and this effect was related to striatal and hippocampal activity as revealed by fMRI (Murty et al., 2015). Therefore, another parsimonious explanation for our null finding is that a stronger sense of agency, possibly associated with the engagement of memory related brain regions, is necessary in order to induce a positive effect of novelty on long-term memory. This hypothesis should be further investigated and has potentially important implications for possible interventions, which would need to include an active novelty manipulation.

A third possible explanation for our null finding relates to the length and onset of the novelty experience. Regarding the length, at least one animal study suggests that a 5-min novelty exploration is most efficient to induce LTP (Li et al., 2003); therefore, in subsequent human studies, novelty was presented for 5 min (Fenker et al., 2008; Schomaker et al., 2014). However, in the aforementioned study by Ballarini et al. (2013), a novel science class before learning was 20 min long; and in a study with human adults, a positive effect of novelty on learning has been shown with several repeating learning contexts that were approx. 6 min long (Bunzeck and Düzel, 2006). Finally, a 15-min stay in a novel environment led to a reinforcement of early- to late-LTP in rats (Straube et al., 2003) further suggesting that a limitation of 5 min might not necessarily be justified. In any case, our 13-min novelty presentation did not promote learning, which leaves the optimal length unclear.

In terms of onset, evidence suggests that a close proximity between novelty and the learning task is important. For instance, a weak high-frequency conditioning stimulation only induced LTP when rats explore a novel environment 5 min before, but not 1 day before stimulation (Li et al., 2003). In humans, a novel science lesson only promoted learning when it was experienced 1 h before or after, but not 4 h before or after reading a story (Ballarini et al., 2013). In our study, novelty experience and learning were close in time, but there was no positive effect on memory. Together with the systematic variation (novelty before and after learning), this suggests that other factors (sense of agency in particular) may more likely explain our null finding.

In our study, recognition memory was tested 1 day after encoding. This delay was based on previous work with a time window of 24 h between encoding and recollection due to the effect of DA on the late phase of LTP (Wittmann et al., 2007; Lisman et al., 2011). However, previous studies also revealed memory improvements by novelty after a short delay (Bunzeck and Düzel, 2006; Schomaker et al., 2014), which leaves it open whether novel movies have an effect on learning right after encoding.

Previous novelty studies differ in the way how memory is being tested. Here, we used a remember/know paradigm in order to differentiate the potential effects of novelty on hippocampus related recollection vs. rhinal cortex-related familiarity. While Schomaker et al. (2014) have used hippocampus-dependent free recall and found a positive effect of novelty on learning, Fenker et al. (2008) could show that free recall and recollection was improved by novelty. Therefore, it appears unlikely that free recall would have revealed a positive effect in our study. However, future studies might include other, more hippocampus-dependent recall and learning tasks, such as spatial navigation, to further pinpoint the exact conditions under which novelty promotes memory.

As expected, RTs were shorter for “remember” as compared to “know” responses (Figure 3). This is in line with previous studies showing that RTs for items that are associated with recollective experiences are typically faster as compared to those without recollective experience (Dewhurst et al., 2006; Rotello and Zeng, 2008; Gimbel and Brewer, 2011). While, at the first glance, this may not be compatible with dual-process models, suggesting that familiarity is a more rapid process than recollection (Jacoby, 1991; Yonelinas, 2002), the slower RTs for “know” responses might reflect difficulties in old judgments without the retrieval of contextual details (Henson et al., 1999); this also fits to the notion of “remember” responses having an all-or-none quality, while “know” responses require a post-retrieval processing to determine their familiarity (Dewhurst and Conway, 1994; Dewhurst et al., 2006). In any case, our findings do not provide evidence that novelty exposure impacts on either form of recognition memory. This has been expected for “remember” responses in particular, given its closer link to the hippocampus (Diana et al., 2007), which receives dopaminergic innervations (Lisman and Grace, 2005). However, our finding must be interpreted with the limitations and possible explanations mentioned above; therefore, they do not rule out that novelty does selectively impact on “remember” responses, for instance, when an active task on the novel material is employed.

Together, novel movie sequences were perceived as more interesting and led to better mood as compared to familiar movies. However, novelty exposure before or after learning a word list did not promote recollection- or familiarity-based recognition memory. This is incompatible with previous studies in humans and animals, which could show a positive effect of novelty exposure on LTP and long-term memory. Our findings suggest that a simple exposure to novelty is not sufficient to promote learning; instead, an active task with the novel stimulus material appears important. This hypothesis has important implications for possible interventions, and, therefore, needs to be tested in future studies.

## Data Availability

All datasets generated for this study are included in the manuscript and/or the supplementary files.

## Ethics Statement

The study was approved by the local ethics committee of the University of Lübeck, Germany, and in accordance with the Declaration of Helsinki.

## Author Contributions

DB acquired the data. DB and NB designed the study, analyzed the data, and wrote the article.

### Conflict of Interest Statement

The authors declare that the research was conducted in the absence of any commercial or financial relationships that could be construed as a potential conflict of interest.

## References

[ref1] Ballarini F. Martínez M. C. Perez M. D. Moncada D. Viola H. (2013). Memory in elementary school children is improved by an unrelated novel experience. PLoS One 8:e66875. 10.1371/journal.pone.0066875, PMID: 23840541 PMC3686730

[ref2] Ballarini F. Moncada D. Martinez M. C. Alen N. Viola H. (2009). Behavioral tagging is a general mechanism of long-term memory formation. Proc. Natl. Acad. Sci. USA 106, 14599–14604. 10.1073/pnas.0907078106 19706547 PMC2732837

[ref500] Brainard D. H. (1997). The Psychophysics Toolbox. Spat Vis. 10, 433–436. 9176952

[ref3] Bunzeck N. Dayan P. Dolan R. J. Duzel E. (2010). A common mechanism for adaptive scaling of reward and novelty. Hum. Brain Mapp. 31, 1380–1394. 10.1002/hbm.20939, PMID: 20091793 PMC3173863

[ref4] Bunzeck N. Düzel E. (2006). Absolute coding of stimulus novelty in the human substantia nigra/VTA. Neuron 51, 369–379. 10.1016/j.neuron.2006.06.021, PMID: 16880131

[ref5] Bunzeck N. Guitart-Masip M. Dolan R. J. Duzel E. (2014). Pharmacological dissociation of novelty responses in the human brain. Cereb. Cortex 24, 1351–1360. 10.1093/cercor/bhs420, PMID: 23307638 PMC3977623

[ref6] Chowdhury R. Guitart-Masip M. Bunzeck N. Dolan R. J. Düzel E. (2012). Dopamine modulates episodic memory persistence in old age. J. Neurosci. 32, 14193–14204. 10.1523/JNEUROSCI.1278-12.2012 23055489 PMC3734374

[ref7] Davis C. D. Jones F. L. Derrick B. E. (2004). Novel environments enhance the induction and maintenance of long-term potentiation in the dentate gyrus. J. Neurosci. 24, 6497–6506. 10.1523/JNEUROSCI.4970-03.2004 15269260 PMC6729872

[ref8] Dewhurst S. A. Conway M. A. (1994). Pictures, images, and recollective experience. J. Exp. Psychol. Learn. Mem. Cogn. 20, 1088–1098. 10.1037/0278-7393.20.5.1088, PMID: 7931096

[ref9] Dewhurst S. A. Holmes S. J. Brandt K. R. Dean G. M. (2006). Measuring the speed of the conscious components of recognition memory: remembering is faster than knowing. Conscious. Cogn. 15, 147–162. 10.1016/j.concog.2005.05.002, PMID: 16019226

[ref10] Diana R. A. Yonelinas A. P. Ranganath C. (2007). Imaging recollection and familiarity in the medial temporal lobe: a three-component model. Trends Cogn. Sci. 11, 379–386. 10.1016/j.tics.2007.08.001, PMID: 17707683

[ref11] Duszkiewicz A. J. McNamara C. G. Takeuchi T. Genzel L. (2019). Novelty and dopaminergic modulation of memory persistence: a tale of two systems. Trends Neurosci. 42, 102–114. 10.1016/j.tins.2018.10.002, PMID: 30455050 PMC6352318

[ref12] Fenker D. B. Frey J. U. Schuetze H. Heipertz D. Heinze H.-J. Duzel E. (2008). Novel scenes improve recollection and recall of words. J. Cogn. Neurosci. 20, 1250–1265. 10.1162/jocn.2008.20086, PMID: 18284351

[ref13] Gimbel S. I. Brewer J. B. (2011). Reaction time, memory strength, and fMRI activity during memory retrieval: hippocampus and default network are differentially responsive during recollection and familiarity judgments. Cogn. Neurosci. 2, 19–23. 10.1080/17588928.2010.513770, PMID: 21278912 PMC3026441

[ref14] Granado N. Ortiz O. Suárez L. M. Martín E. D. Ceña V. Solís J. M. (2008). D1 but not D5 dopamine receptors are critical for LTP, spatial learning, and LTP-induced arc and zif268 expression in the hippocampus. Cereb. Cortex 18, 1–12. 10.1093/cercor/bhm026 17395606

[ref15] Greiner B. (2015). Subject pool recruitment procedures: organizing experiments with ORSEE. J. Econ. Sci. Assoc. 1, 114–125. 10.1007/s40881-015-0004-4

[ref16] Henson R. N. A. Rugg M. D. Shallice T. Josephs O. Dolan R. J. (1999). Recollection and familiarity in recognition memory: an event-related functional magnetic resonance imaging study. J. Neurosci. 19, 3962–3972. 10.1523/JNEUROSCI.19-10-03962.1999, PMID: 10234026 PMC6782715

[ref17] Herweg N. A. Sommer T. Bunzeck N. (2018). Retrieval emands adaptively change striatal old/new signals and boost subsequent long-term memory. J. Neurosci. 38, 745–754. 10.1523/JNEUROSCI.1315-17.2017, PMID: 29217684 PMC6596196

[ref18] Jacoby L. L. (1991). A process dissociation framework: separating automatic from intentional uses of memory. J. Mem. Lang. 30, 513–541. 10.1016/0749-596X(91)90025-F

[ref19] Kempadoo K. A. Mosharov E. V. Choi S. J. Sulzer D. Kandel E. R. (2016). Dopamine release from the locus coeruleus to the dorsal hippocampus promotes spatial learning and memory. Proc. Natl. Acad. Sci. USA 113, 14835–14840. 10.1073/pnas.1616515114, PMID: 27930324 PMC5187750

[ref20] Li S. Cullen W. K. Anwyl R. Rowan M. J. (2003). Dopamine-dependent facilitation of LTP induction in hippocampal CA1 by exposure to spatial novelty. Nat. Neurosci. 6, 526–531. 10.1038/nn1049, PMID: 12704392

[ref21] Lisman J. E. Grace A. A. (2005). The hippocampal-VTA loop: controlling the entry of information into long-term memory. Neuron 46, 703–713. 10.1016/j.neuron.2005.05.002, PMID: 15924857

[ref22] Lisman J. Grace A. A. Duzel E. (2011). A neoHebbian framework for episodic memory; role of dopamine-dependent late LTP. Trends Neurosci. 34, 536–547. 10.1016/j.tins.2011.07.006, PMID: 21851992 PMC3183413

[ref23] McNamara C. G. Dupret D. (2017). Two sources of dopamine for the hippocampus. Trends Neurosci. 40, 383–384. 10.1016/j.tins.2017.05.005, PMID: 28511793 PMC5489110

[ref24] Moncada D. Viola H. (2007). Induction of long-term memory by exposure to novelty requires protein synthesis: evidence for a behavioral tagging. J. Neurosci. 27, 7476–7481. 10.1523/JNEUROSCI.1083-07.2007 17626208 PMC6672624

[ref25] Murty V. P. DuBrow S. Davachi L. (2015). The simple act of choosing influences declarative memory. J. Neurosci. 35, 6255–6264. 10.1523/JNEUROSCI.4181-14.2015, PMID: 25904779 PMC4405547

[ref26] O’Carroll C. M. Morris R. G. M. (2004). Heterosynaptic co-activation of glutamatergic and dopaminergic afferents is required to induce persistent long-term potentiation. Neuropharmacology 47, 324–332. 10.1016/j.neuropharm.2004.04.005, PMID: 15275821

[ref27] Packard M. G. White N. M. (1991). Dissociation of hippocampus and caudate nucleus memory systems by posttraining intracerebral injection of dopamine agonists. Behav. Neurosci. 105, 295–306. 10.1037/0735-7044.105.2.295, PMID: 1675062

[ref28] Park J. Shimojo E. Shimojo S. (2010). Roles of familiarity and novelty in visual preference judgments are segregated across object categories. Proc. Natl. Acad. Sci. USA 107, 14552–14555. 10.1073/pnas.1004374107 20679235 PMC2930416

[ref29] Rotello C. M. Zeng M. (2008). Analysis of RT distributions in the remember—know paradigm. Psychon. Bull. Rev. 15, 825–832. 10.3758/PBR.15.4.825, PMID: 18792511

[ref30] Schomaker J. Meeter M. (2015). Short- and long-lasting consequences of novelty, deviance and surprise on brain and cognition. Neurosci. Biobehav. Rev. 55, 268–279. 10.1016/j.neubiorev.2015.05.002, PMID: 25976634

[ref31] Schomaker J. van Bronkhorst M. L. V. Meeter M. (2014). Exploring a novel environment improves motivation and promotes recall of words. Front. Psychol. 5:918. 10.3389/fpsyg.2014.00918, PMID: 25191297 PMC4138787

[ref32] Straube T. Korz V. Balschun D. Frey J. U. (2003). Requirement of beta-adrenergic receptor activation and protein synthesis for LTP-reinforcement by novelty in rat dentate gyrus. J. Physiol. 552, 953–960. 10.1113/jphysiol.2003.049452, PMID: 12937286 PMC2343450

[ref33] Takeuchi T. Duszkiewicz A. J. Sonneborn A. Spooner P. A. Yamasaki M. Watanabe M.. (2016). Locus coeruleus and dopaminergic consolidation of everyday memory. Nature 537, 357–362. 10.1038/nature19325, PMID: 27602521 PMC5161591

[ref34] Tulving E. (1985). Memory and consciousness. Can. Psychol. 26, 1–12. 10.1037/h0080017

[ref35] Wang S.-H. Redondo R. L. Morris R. G. M. (2010). Relevance of synaptic tagging and capture to the persistence of long-term potentiation and everyday spatial memory. Proc. Natl. Acad. Sci. USA 107, 19537–19542. 10.1073/pnas.1008638107 20962282 PMC2984182

[ref36] Wittmann B. C. Bunzeck N. Dolan R. J. Düzel E. (2007). Anticipation of novelty recruits reward system and hippocampus while promoting recollection. NeuroImage 38, 194–202. 10.1016/j.neuroimage.2007.06.038, PMID: 17764976 PMC2706325

[ref37] Yonelinas A. P. (2002). The nature of recollection and familiarity: a review of 30 years of research. J. Mem. Lang. 46, 441–517. 10.1006/jmla.2002.2864

[ref38] Yonelinas A. P. Aly M. Wang W.-C. Koen J. D. (2010). Recollection and familiarity: examining controversial assumptions and new directions. Hippocampus 20, 1178–1194. 10.1002/hipo.20864, PMID: 20848606 PMC4251874

[ref39] Yonelinas A. P. Dobbins I. Szymanski M. D. Dhaliwal H. S. King L. (1996). Signal-detection, threshold, and dual-process models of recognition memory: ROCs and conscious recollection. Conscious. Cogn. 5, 418–441. 10.1006/ccog.1996.0026, PMID: 9063609

[ref40] Zaehle T. Bauch E. M. Hinrichs H. Schmitt F. C. Voges J. Heinze H.-J.. (2013). Nucleus accumbens activity dissociates different forms of salience: evidence from human intracranial recordings. J. Neurosci. 33, 8764–8771. 10.1523/JNEUROSCI.5276-12.2013, PMID: 23678119 PMC6618843

